# Medical-Grade Honey Outperforms Conventional Treatments for Healing Cold Sores—A Clinical Study

**DOI:** 10.3390/ph14121264

**Published:** 2021-12-04

**Authors:** Piyu Parth Naik, Dimitris Mossialos, Bas van Wijk, Petra Novakova, Frank A. D. T. G. Wagener, Niels A. J. Cremers

**Affiliations:** 1 Saudi German Hospitals and Clinics, Department of Dermatology, Hessa Street 331 West, Al Barsha 3, Exit 36 Sheikh Zayed Road, Dubai P.O. Box 391093, United Arab Emirates; drpiyu85@gmail.com; 2 Department of Biochemistry and Biotechnology, University of Thessaly, Biopolis-Mezurlo, 41500 Larisa, Greece; mosial@bio.uth.gr; 3 Department of Dentistry, Radboud University Medical Center, Philips van Leydenlaan 25, 6525 EX Nijmegen, The Netherlands; bas.vanwijk@radboudumc.nl (B.v.W.); frank.wagener@radboudumc.nl (F.A.D.T.G.W.); 4 LERAM Pharmaceuticals s.r.o., Páteřní 1216/7, 63500 Brno, Czech Republic; petra.novakova@leram-pharma.cz; 5 Triticum Exploitatie BV, Sleperweg 44, 6222 NK Maastricht, The Netherlands

**Keywords:** medical-grade honey, herpes simplex virus, cold sores, wounds, pain, itch

## Abstract

Cold sores are nasolabial blisters caused by herpes simplex virus (HSV) infections. Novel therapies demonstrating simultaneously antiviral activity and improved wound healing are warranted. The aim of this study was to investigate the efficacy of medical-grade honey (MGH) for treating HSV-induced cold sores. A crossover trial was performed in patients with recurrent cold sores (*n* = 29). The majority (65.6%) of these patients experience four or more episodes per year, thus forming a valid self-control group. In this study, patients applied an MGH-based formulation (L-Mesitran Soft) on their cold sore at the onset of symptoms (62.1%) or appearing of blister (37.9%) and compared it to their conventional treatments. After complete healing, patients filled in a questionnaire evaluating healing, pain, and itching. The average absolute healing time was 72.4% slower with conventional treatment (10.0 days) compared to MGH (5.8 days). After MGH treatment, 86.2% of all patients experienced faster objective healing (6.9% similar and 6.9% slower) and the subjective healing score was higher in 79.3% of the patients (20.7% similar). If the patients normally experience pain and itching during their cold sores, these levels were lower with MGH therapy compared to conventional treatment in 72.7% and 71.4% of the patients, respectively. Moreover, 100% of the patients prefer MGH treatment over conventional treatment and will use it again on future cold sores. MGH is a promising alternative treatment for cold sores, likely by combining both increased antiviral and wound healing activities while alleviating pain and itching.

## 1. Introduction

Cold sores are nasolabial blisters caused by herpes simplex virus (HSV) infections. Approximately two-thirds of the global population between 0 and 49 years of age have HSV-1 infection, accounting for an estimated 3.7 billion people [1,2]. Most of these infections are oral, while 122 to 192 million people are estimated to have genital HSV-1 infection [1]. HSV-2 causes genital infections which are less prevalent (11% globally) [3]. However, the incidence of cold sores is considerably lower than the infection rates, as the infection is often present in a latent form. The average incidence is 1.6 per 1000 patients per year and its prevalence is 2.5 per 1000 patients per year [4,5]. Approximately one-third of all infected patients suffer relapses [4,5,6,7]. Although most individuals with recurrent cold sores have up to two episodes in an entire year, 5–10% has at least six recurrences per annum [7,8]. Triggering elements of recurrent cold sores comprise stress, illness, premenstrual tension, ultra-violet light, or surgical procedures. Moreover, having a compromised antiviral response, such as patients with atopic dermatitis harboring fillagrin mutations may enhance these triggering events [8,9].

**Figure 1:**
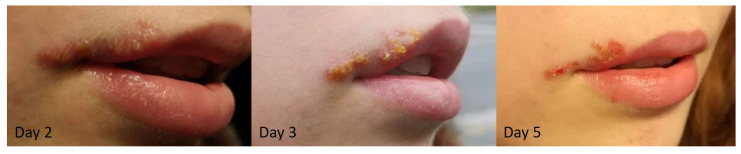
Example of the development of a cold sore, from the vesicular stage (**left**) to blister burst and drying out (**center**) to scab formation (**right**).

Cold sores develop via several stages characterized by different symptoms (Figure 1). In the first stage, patients may feel tingling, itching, or a burning sensation, but without the blister being formed yet. Next, peri-oral and intra-oral lesions will develop, starting with the vesicular stage (1–2 days after initial symptoms), the opening of the blister(s) (around day 4), and subsequently drying out (day 5–8), leaving a yellow or brown crust, which eventually flakes off. The final stage is the healing, and scabs will become smaller in time until complete healing (normally 7–14 days) [7,10,11]. Effective treatments options are limited and often restrain on antiviral drugs and topical creams, such as acyclovir (Zovirax), famciclovir (Famvir), valacyclovir (Valtrex), docosanol (Abreva), and penciclovir (Denavir) limiting viral DNA replication [7,12]. These treatments may reduce the duration of a cold sore by 0.5 to 2.5 days, depending on the type of medication and dosage used [4,12]. However, these drugs may cause in some cases (1–10%) various side effects, including discouragement, irritability, tiredness, nausea, vomiting, cramps, diarrhea, body aches or pain, rash, itching, and sore throats [13,14,15]. Clinicians also prescribe nonsteroidal anti-inflammatory drugs (NSAIDs) to reduce associated pain [16,17,18,19]. Cold sores are uncomfortable, negatively affect aesthetics, and can be considered embarrassing, creating a psychosocial impact that affects the quality of life of these patients (Figure 1) [8,19]. Novel therapies that have antiviral activity with fewer side effects and decreased wound healing time are eagerly awaited.

Natural products may offer such a promising treatment. Medical-grade honey (MGH) exerts a broad range of antimicrobial activities via multiple mechanisms, including low pH, strong osmotic actions, production of hydrogen peroxide, and the presence of antimicrobial molecules [20].

The antimicrobial activity of honey against bacteria and fungi has been extensively documented in the literature; however, the antiviral activity of MGH is less described [21,22]. MGH exerts in vitro antiviral activity against multiple viruses, including HSV-1, varicella zoster virus, and influenza virus A (H1N1) [23,24,25]. Interestingly, a recent systematic review and meta-analysis evaluating 14 randomized controlled trials concluded that honey was superior to usual care for the improvement of symptoms of upper respiratory tract infections and provides a widely available and cheap alternative to antibiotics [26]. Honey also forms a promising potent treatment for COVID-19 patients and clinical trials are ongoing [27,28,29]. A clinical randomized-controlled trial showed that honey in combination with *Nigella sativa* consumption by COVID-19 patients led to faster clearance of the virus and relief of symptoms, earlier hospital discharge, and a four-fold lower mortality rate in severe cases [30].

Synergistic antimicrobial activity of honey and vitamins C and E have previously been demonstrated. Vitamin C and E supplements in the MGH-based wound care formulation (L-Mesitran Soft) enhance the antimicrobial and wound healing activities when compared to the raw honey component [31,32,33,34,35]. Since both honey and vitamins C and E have been shown to possess antiviral activity [36,37,38,39,40], they may also help to treat cold sores. The wound healing activities of MGH are also based on multiple mechanisms, and therefore may further advance the healing of the cold sores [41,42].

The aim of the present study was to investigate whether topical application of the MGH-based formulation L-Mesitran Soft would be an effective alternative treatment for treating recurrent cold sores when compared to conventional treatments. The working hypothesis was that MGH exerts antiviral activity, promotes wound repair, and improves the patient’s quality of life.

## 2. Results

### 2.1. Healing Time Is Lower with MGH Compared to Conventional Treatment

**Figure 2:**
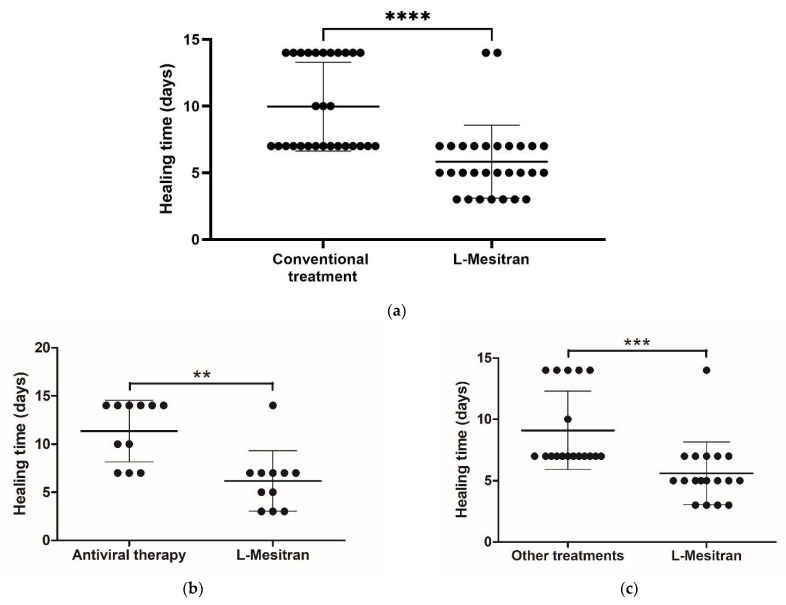

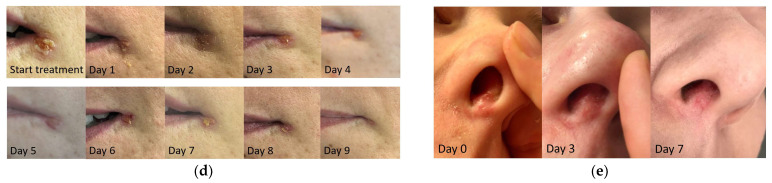
L-Mesitran reduces the healing time of cold sores compared to conventional treatment. (**a**) Dotplot of absolute healing time in days for both conventional and L-Mesitran treatment, each dot represents one patient (*n* = 29). (**b**) Dot plot of absolute healing time of the subpopulation who use antiviral acyclovir-based therapy (Zovirax, herpesin, or vectavir). (**c**) Dot plot of absolute healing time of the subpopulation who use other treatments and do not use antiviral therapy as conventional treatment. (**d**) Representative example labial cold sore. Normally, with conventional treatments healing takes 14 days, but healing was achieved within 9 days with L-Mesitran. (**e**) Representative example nasal cold sore. Normally, healing takes 14 days, but with L-Mesitran it was healed in 7 days. Significant differences between the groups are indicated by (** p < 0.01, *** p < 0.001, **** p < 0.0001).

Patients were asked via a questionnaire how long it normally takes for the cold sores to completely heal following conventional treatment, and how long it took with MGH treatment. The average absolute healing time was significantly higher (*p* < 0.0001) with conventional treatments (10.0 days) compared to MGH (treatment (5.8 days) (Figure 2a). On average, this corresponds to slower healing of 72.4% (10.0/5.8) with conventional treatment compared to MGH treatment.

A sub-analysis could be performed because a large subpopulation of the patients (11/29 = 37.9%) use antiviral therapy as a conventional treatment. In this sub-analysis, patients whose conventional treatments consist of acyclovir-based drugs (Zovirax, Herpesin, or Vectavir) and patients using other treatments (18/29 = 62.1%) were analyzed separately. For both subpopulations, it was investigated whether there is a difference in absolute healing time between their conventional treatment and MGH treatment (Figure 2b,c). The average absolute healing time within the subpopulation using antiviral therapy was 5.2 days shorter (*p* = 0.001) with MGH, 6.2 days versus 11.4 days (Figure 2b). The average healing time within the other subpopulation that uses other treatments was 3.5 days shorter (*p* = 0.0009) with MGH, 5.6 days versus 9.1 days (Figure 2c).

Based on the absolute healing times, the objective healing time was determined for the complete patient population. The objective healing showed that 86.2% (25/29) of the patients experienced significantly faster healing (*p* < 0.005), while 6.9% had similar or slower healing (2/29 each) (Table 1). Moreover, patients were asked about their subjective feeling if healing was faster or not with MGH. These results were in line with the objective healing times and 79.3% (23/29) of the patients achieved faster subjective healing with MGH compared to their conventional treatments, while healing was experienced similar in 20.7% (6/29), and none of them experienced slower healing (0/29) (Table 1). Representative examples of the progression of labial and nasal cold sores with L-Mesitran treatment are shown in Figure 2d,e.

### 2.2. Pain Is Lower with MGH Than Conventional Treatment

Not all patients experience pain during their cold sore episodes. In our patient group, 22 out of 29 patients (75.9%) normally experience pain. Within this group, 72.7% of the patients (16/22) experience less pain with MGH, 27.3% of the patients (6/22) experiences a similar pain level and none of the patients (0/22) experiences more pain (Table 2).

### 2.3. Less Itching with MGH Than Conventional Treatment

Not all patients experience an itching sensation during their episode of cold sores. In our patient group, 21 out of 29 patients (72.4%) normally experience itching. Within this group, 71.4% of the patients (15/21) experience less itching, 28.6% (6/21) experience a similar level of itching, and none of the patients (0/21) experiences more itching (Table 3).

### 2.4. Patients Strongly Prefer MGH over Conventional Therapies

Based on their positive experiences, all included patients (29/29) will use L-Mesitran for the treatment of future cold sores. Most patients experienced a benefit on multiple parameters, (faster objective healing, faster subjective healing, less pain, and less itching). 93.1% (27/29) of the patients experienced a beneficial effect on at least one of these parameters, 82.8% (24/29) had a beneficial effect on at least two parameters, 62.1% (18/29) on at least three parameters, and 34.5% (10/29) experienced a benefit on all four parameters. Notably, many patients do not normally experience pain or itching and therefore this score is under representative.

The open feedback from the patients was also very positive (Supplementary Table S1). Most of them refer to the faster healing or reduction of symptoms. However, many patients also liked the hydrating effect and expressed that “the skin is more elastic, softer, dries out less, and the blisters are less prone to rupture/crack”. Moreover, multiple patients also liked the formulation itself; “staying well in place after application, the color of the product being invisible on the lip, and its taste”. However, some patients also disliked the taste as they do not like honey. Interestingly, one of the patients who normally developed cold sores 4–5 times per year started applying L-Mesitran twice daily as a preventive measure, and she did not develop a new cold sore since then for eight months (still ongoing).

## 3. Discussion

Cold sores are caused by HSV1 and HSV2 and cause problems such as itching, pain, and cosmetic appearance. Conventional treatments focus solely on antiviral activity; however, they do not address the wound that follows the viral infection. The MGH-based formulation (L-Mesitran Soft) has broad-spectrum antimicrobial and antiviral activity and promotes wound repair. It was therefore postulated to form an attractive novel therapy to treat cold sores. In this trial, the average healing time was significantly reduced from 10.0 days with conventional treatments to 5.8 days with MGH. The absolute healing time of the subpopulations using antiviral therapy and other treatments was in line with the results of the total population. Thus, the reduced healing time with MGH was irrespective of the type of the previously used conventional treatment. Moreover, symptoms as pain and itching were reduced with MGH in 72.7% and 71.4% of the patients, respectively, when compared to conventional treatments.

Previous studies investigating the effect of the antiviral acyclovir cream observed a reduction in healing time in eight out of ten trials, varying from 0.5 (4.3 vs. 4.816) to 2.5 (5.7 vs. 8.315) days [4]. None of these studies reported a decrease in the duration or severity of pain following acyclovir treatment. Two studies investigating penciclovir showed similar effects, however, one of these studies also reported a reduction in pain duration by penciclovir (3.5 vs. 4.1 days) [4].

A reduction of 4.2 days with MGH compared to conventional treatments and reduction in pain and itching is therefore very impressive. However, this may be simply explained by MGH having both antimicrobial/antiviral and wound healing activities. MGH has in vitro antiviral activity against multiple viruses, including HSV-1, varicella zoster virus, and influenza virus A (H1N1), and showed clinical efficacy against upper respiratory tract infections and, possibly, COVID-19 [23,24,25,26,27,28,29]. Its wound healing properties are orchestrated by multiple mechanisms. It creates a moist wound environment, promotes autolytic debridement, angiogenesis, and re-epithelialization, and reduces oxidative and inflammatory stress [31,41,43,44,45,46]. Moreover, MGH has previously been shown to have anti-inflammatory activity, which is in line with the reduced pain and itching in the current study [41,43]. Drying and cracking make the lesions more susceptible to secondary bacterial infections; the hydrating properties of honey can inhibit bacterial infections by preventing cracking, but also via its potent antimicrobial activities [42,47].

Three studies investigated the effect of honey on cold sore treatment before. A recent randomized controlled trial in 952 human patients with cold sores showed that there was no difference in efficacy between topical medical-grade kanuka honey (90% honey with 10% glycerine) cream and 5% acyclovir cream [48]. The median time to return to normal skin was between 8 and 9 days for both acyclovir and honey groups (*p* = 0.56), and there was no difference in pain level (*p* = 0.56) and duration (*p* = 0.90) [48]. In another cross-over trial, the effect of the topical application of honey in 16 patients with recurrent attacks of labial and genital herpes lesions was investigated and compared to acyclovir cream [49]. The used honey had a multifloral origin and was gathered in the United Arab Emirates and dark yellow in color. The mean duration of pain and mean healing time with honey treatment were 39% and 43% better for labial herpes and 50% and 59% better for genital herpes, respectively, when compared to acyclovir [49]. Honey reduced healing time by 3.28 days for labial herpes and 2.57 days for genital herpes compared to acyclovir [49]. The third trial, a randomized double-blind placebo-controlled study examined the combined efficacy of commercially or locally (Egypt) available honey with acyclovir suspension compared to acyclovir alone in one hundred children aged 2–8 years. The combination of honey with acyclovir led to faster disappearance of the lesion (3 days vs. 6 days), fewer eating difficulties, and lower pain scores (all significant) [50].

The latter two studies are well in line with our findings, showing that MGH-based L-Mesitran Soft was highly effective. The difference in efficacy with the first study may lay in the different types of honey being used. Methylglyoxal-based honey (such as kanuka) has previously been demonstrated to be less effective for the treatment of mucositis than hydrogen peroxide-based honey (such as the honey used in the latter two studies and the L-Mesitran formulation) [51,52]. In addition, the vitamin supplements in L-Mesitran have likely further enhanced the antiviral and healing activity of MGH, making it more effective than current standard therapies. This synergistic antimicrobial activity of MGH with the supplements was previously demonstrated against multiple bacteria and fungi [32,33,34,35,53]. Instead of a randomized controlled trial in which two groups received different treatments, our study was performed on patients with recurrent cold sores, and their historic experience with other conventional treatments was used as a control arm. Patients with recurrent cold sores have extensive experience with treating their cold sores since their outcome is based on multiple episodes and different treatments, this may provide a more reliable within-patient matched control group.

Interestingly, one of the patients in our study kept using L-Mesitran as a preventive measure after treatment of her cold sore was successful and she did not develop cold sores for eight months (still ongoing), even not when getting the flu. More research on preventive therapies would be interesting to follow up. In a study among 147 skiers with a history of sun-induced recurrences, oral administration of acyclovir starting 12 days before sun exposure showed a reduced number of lesions 7% (5 out of 75 acyclovir-treated patients) compared with 26% (19 out of 72 placebo-treated patients) [54]. A setting in which patients have an increased chance to develop cold sores, such as in skiers, would be perfect to investigate the prophylactic activity of MGH on cold sore recurrence. Recently, MGH (L-Mesitran Soft) demonstrated prophylactic activity against infections in two randomized controlled studies. A single subcutaneous application in lacerations and after colic surgeries in horses decreased infection rates a 3-and 4-fold, respectively [55,56]. Complete healing of lacerations was also improved, supporting another important asset: promoting wound healing [56]. These activities suggest that L-Mesitran could serve as a putative new therapy for treating cold sores. However, more research into the exact mechanisms and the prophylactic activity is needed.

## 4. Materials and Methods

### 4.1. Design of the Study

A crossover trial was performed in patients suffering from recurrent cold sores. In our study, 65.5% of the participants suffer from four or more cold sore episodes per year (Table 4). These patients have thus extensive experience in treating their cold sores and serve as the best matched-control group. This study design allowed within-patient comparisons between treatments because they can serve as their own control subject, removing the inter-patient variability from the comparison between the treatments and removing bias between different patients’ experiences. Disease pathogenesis and prognosis are dependent on a combination of host-and virus-specific factors and may hence differ more between different patients than within recurrent episodes within the same patient [11,57]. Moreover, since patients included in this study have already optimized their personal treatment over time they can deliver first-hand experiences of their earlier treatments against cold sores. Since cold sores come in ‘episodes’, there is no carry-over effect and a washout period is not needed.

### 4.2. Patient Population

Inclusion took place in two ways; patients with recurrent cold sores were recruited via a social media campaign or when presenting to the dermatologist. The recruitment via social media was carried out via Facebook, and patients with recurrent cold sores applied for participation. There were three calls, in which a total of 56 people responded. All of them received a free tube of L-Mesitran Soft via regular mail and they subsequently waited for a cold sore to develop. After one year since the first call, we decided to terminate the inclusion. All patients who developed a cold sore were asked to fill in a questionnaire to investigate the experience of the patients (31 patients). Only adult patients who had recurrent cold sores and who completed the treatment were included (excluding two patients). In total, 29 patients were included in the study, of which four males and 25 females. The age of the patients was classified over different ranges (Table 5). Moreover, patients were asked about the frequency they develop cold sores per year (Table 4), showing that most patients in this study often experience recurrent cold sores.

### 4.3. Treatment

The participating patients were asked to apply L-Mesitran Soft (an MGH-based formulation, manufactured by Theo Manufacturing, Maastricht, The Netherlands) on their cold sores as monotherapy three times daily. L-Mesitran Soft is a medical device class 2B (FDA approved and CE certified) and used for wound care. L-Mesitran Soft consists of MGH (40%), lanolin, PEG4000, propylene glycol, and vitamins C and E, and adheres to strict standards to ensure its quality, safety, and efficacy [20].

Interestingly, all included patients normally use “some kind of treatment” and none of them leave their cold sores untreated. Conventional treatments were very diverse and 37.9% of the patients (11 out of 29) commonly used multiple products. Most of the patients normally treat their cold sores with antiviral therapy, such as Zovirax (acyclovir), herpesin (acyclovir-based cream) or vectavir (penciclovir) (11/29 = 37.9%), followed by herbal products (4/29 = 13.8%), tea tree-based products (3/29 = 10.3%), hemagel (wound care product) (3/29 = 10.3%), zinc-based cream (3/29 = 10.3%), lip balm (2/29 = 6.9%), vitamin B-based products (2/29 = 6.9%), an “unspecified” product from the pharmacy (2/29 = 6.9%), or other products (herpes patch, Urgo Film, vaseline, betadine, bepanthen, Bactroban, L-lysine).

### 4.4. Outcome Parameters

Patients were asked to fill in an online questionnaire after their cold sores healed to obtain basic patient information and to assess their experience with L-Mesitran Soft. The questionnaire (Supplementary Table S2) consisted of 15 questions and all fields in the questionnaire were mandatory to be filled in. The answers to these questions were used to determine the following outcomes: absolute healing time (shown as the number of days needed to heal for each treatment) and objective healing time (as a measure of absolute healing time of conventional treatment compared to the absolute healing time with L-Mesitran Soft (Questions 7 and 10), subjective healing time (Question 9), pain experience (Question 11), itching experience (Question 12), future use (Question 14). Moreover, patients had the opportunity to submit photos of their cold sores to monitor the healing progression. This was conducted by 11 patients (37.9%).

### 4.5. Ethical Statement

Ethical approval to perform the study was granted (reference number DSREC-09/2021_07) by the Dubai Health Authority. The patients were informed about the study, and they all gave electronic informed consent to participate in the study and publication of the data and, when provided, photos. The principles of the World Medical Association’s Declaration of Helsinki were followed.

### 4.6. Statistics

The data presented in Figure 2a (dot plot of the absolute healing time) were analyzed with the Mann–Whitney test using GraphPad Prism software, version 8.01 (San Diego, CA, USA). Results were considered significant different when *p* < 0.05 (* *p* < 0.05, ** *p* < 0.01, *** *p* < 0.001) **** *p* < 0.0001). The other parameters, such as subjective healing time, pain, and itching were analyzed using the paired-samples sign test with an equal allocation of ties.

## 5. Conclusions

Topical application of the MGH-formulation L-Mesitran Soft on cold sores was very effective. In this patient population with recurrent cold sores, the objective and subjective healing time was decreased in 86.2% and 79.3% of the patients when compared to conventional treatments, respectively, and resulted in reducing the healing time from 10.0 to 5.8 days. Moreover, more than 70% of the affected patients experienced less pain and itching. Together, MGH strongly improves the quality of life of the patients. L-Mesitran is a potent alternative treatment for treating cold sores, likely due to the combination of antiviral and wound healing activities.

## Figures and Tables

**Table 1:** Efficacy MGH treatment on the objective and subjective healing time when compared to conventional treatments.

	Number of Patients Evaluated (% of Cohort)	Faster Healing with MGH Number (%)	Similar Healing Number (%)	Slower Healing with MGH Number (%)	Significant Different Compared to Conventional Treatment
Objective healing time	29 (100)	25 (86.2)	2 (6.9)	2 (6.9)	*p* < 0.005
Subjective healing experience	29 (100)	23 (79.3)	6 (20.7)	0 (0)	*p* < 0.005

**Table 2:** Effect treatment on the experience of pain.

	Number of Patients Experiencing Pain (% of Cohort)	Less Pain with MGH Number (%)	Similar Pain Number (%)	More Pain with MGH Number (%)	Significance
MGH treatment compared to conventional treatment	22 (75.9)	16 (72.7)	6 (27.3)	0 (0)	*p* < 0.005

**Table 3:** Relative itching when comparing MGH treatment with conventional treatments.

	Number of Patients Experiencing Itching (% of Cohort)	Less Itching with MGH Number (%)	Similar Itching Number (%)	More Itching with MGH Number (%)	Significance
MGH treatment compared to conventional treatment	21 (72.4)	15 (71.4)	6 (28.6)	0 (0)	*p* < 0.005

**Table 4:** Distribution of the frequency of the cold sores per patient per year.

Frequency	Quite Exceptionally (about 1 Time per Year)	Occasionally (2–3 Times per Year)	Often (4–5 Times per Year)	Very Often (>5 Times per Year)
Number of patients (total 29)	3	7	10	9
Percentage	10.3%	24.1%	34.5%	31.0%

**Table 5:** Age distribution among participating patients.

Age Group	18–26 Years	27–35 Years	36–44 Years	45–53 Years	54–65 Years	>65 Years
Number of patients (total 29)	8	5	10	3	2	1
Percentage	27.6%	17.2%	34.5%	10.3%	6.9%	3.5%

## Data Availability

All data relevant to the study are included in the article.

## Supplementary Materials

The following are available online at https://www.mdpi.com/article/10.3390/ph14121264/s1, Table S1: Open feedback from the patients, Table S2: Questionnaire for participating cold sore patients.
