# Supplementary material for: Medical-Grade Honey Outperforms Conventional Treatments for Healing Cold Sores—A Clinical Study
Source: Pharmaceuticals (Basel). 2021 Dec 4;14(12):1264. doi: 10.3390/ph14121264 (PMC8706154; doi:10.3390/ph14121264)
Supplement: Supplementary file 1 [file pharmaceuticals-14-01264-s001.zip › pharmaceuticals-1473589-supplementary.pdf]

Supplemental Table S1. Open feedback from the patients.

#*	**Open response**
1	Very quickly healing blisters, the mucous membranes do not dry out so much
2	I will never have time for the initial phase, the cold sores have been working on me overnight. That's why creams do not help much with cold sores. But L-Mesitran helped even though a blister was created. Instead of the usual two weeks, he was healed in less than a week. And especially without a visible scar
3	It is beautifully oily and the skin is more elastic faster
4	There was no swollen lip, the haze soon began to close
5	Advantage: from the first redness to the second day almost complete healing, without blisters. Disadvantage: honey taste is not good
6	The treatment was faster, the lips softer.
7	It is clear, almost invisible on the lip, has a pleasant taste
8	Faster healing
9	lubricates and the skin heals better, better than herpesin
10	color, it is not a white ointment, yellow makes the lip less visible
11	It does not itch and calms
12	Herpes dry faster than with other treatments
13	For me, absolutely great for every blow, abrasion and now even cold sores
14	Relatively large package in a tube. It will suffice for a very long time in the case of the treatment of cold sores. Some may have an unpleasant taste. Personally, I don't like honey, but it's nothing that can't be endured
15	To maintain a larger coat of cream, I need to cover the herpes with a patch
16	The skin of the herpes does not crack
17	The blister is less prone to rupture
18	It is thicker than Hemagel and holds better.
19	Probably hypersensitivity or allergy to any of the ingredients. I had to stop the treatment after the third application.
20	Mesitran works fast! I apply Mesitran twice a day as preventive measure. No complaints or new episodes since then.
21	The treatment was faster and itching was less
22	The treatment was faster and itching was not there
23	No pain and no itch

* 6 out of 29 patients did not gave a response to this question

Supplemental Table S2. Questionnaire for participating cold sore patients.

Questions	Answer options
1. What is your email address?	Open answer
2. Please select a gender	Male/ female
3. Please select an age group	18-26/ 27-35/ 36-44/ 45-53/ 54-65/ >65 (years)
4. How often do you have cold sores?	Quite exceptionally (about 1 time per year)/ Occasionally (2-3 times per year)/ Often (4-5 times per year)/ Very often (more than 5 times per year)
5. What is the location of your cold sore?	On or around the lip/ nasal mucosa/ elsewhere
6. At what stage do you start treatment for cold sores?	After the first symptom (usually itching or tingling)/ when blisters appear/ when scabs are formed/ other
7. How long does it usually take to treat herpes from the first symptoms to healing?	Within 3 days/ less than a week (about 5 days)/ one week (7 days)/ two weeks (14 days)/ more than 14 days/ other
8. What products do you normally use to treat cold sores?	Open answer
9. In your experience, is healing with L-Mesitran faster than other medications used?	Yes/ no/ similar
10. How long does healing with L-Mesitran take?	Within 3 days/ less than a week (about 5 days)/ one week (7 days)/ two weeks (14 days)/ more than 14 days/ other
11. Does L-Mesitran reduce cold sore pain? How would you compare it to other treatments	The pain is the same as with other treatments/ the pain is less with L-Mesitran/ the pain is higher with L-Mesitran/ I do not experience pain when I have a cold sore
12. Does L-Mesitran reduce the itching caused by cold sores? How would you compare it to other treatments.	The itch is the same as with other treatments/ the itch is less with L-Mesitran/ the itch is higher with L-Mesitran/ I do not experience itch when I have a cold sore
13. Based on your experience, please list other advantages or disadvantages of L-Mesitran treatment.	Open answer
14. Based on your experience, will you use L-Mesitran to treat cold sores in the future?	Yes/ no
15. I hereby approve the inclusion of my answers in the questionnaire in the survey results and grant the researchers consent to the possible presentation of the results at medical congresses or in scientific publications. If you agree, we declare that all data will be used anonymously. If you provided photos, the face will be made unrecognizable in all images.	Yes, I agree/ No, I disagree
